# Individual and work-related risk factors for musculoskeletal pain: a cross-sectional study among Estonian computer users

**DOI:** 10.1186/1471-2474-15-181

**Published:** 2014-05-28

**Authors:** Kristel Oha, Liina Animägi, Mati Pääsuke, David Coggon, Eda Merisalu

**Affiliations:** 1 North Estonia Medical Centre, J. Sütiste tee 19, 13419 Tallinn, Estonia; 2 Department of Public Health, University of Tartu, Tartu, Estonia; 3 Tartu Health Care College, Tartu, Estonia; 4 Institute of Exercise Biology and Physiotherapy, University of Tartu, Tartu, Estonia; 5 MRC Lifecourse Epidemiology Unit, University of Southampton, Southampton, UK

**Keywords:** Computer workers, Musculoskeletal pain, Risk factors, CUPID study

## Abstract

**Background:**

Occupational use of computers has increased rapidly over recent decades, and has been linked with various musculoskeletal disorders, which are now the most commonly diagnosed occupational diseases in Estonia. The aim of this study was to assess the prevalence of musculoskeletal pain (MSP) by anatomical region during the past 12 months and to investigate its association with personal characteristics and work-related risk factors among Estonian office workers using computers.

**Methods:**

In a cross-sectional survey, the questionnaires were sent to the 415 computer users. Data were collected by self-administered questionnaire from 202 computer users at two universities in Estonia. The questionnaire asked about MSP at different anatomical sites, and potential individual and work related risk factors. Associations with risk factors were assessed by logistic regression.

**Results:**

Most respondents (77%) reported MSP in at least one anatomical region during the past 12 months. Most prevalent was pain in the neck (51%), followed by low back pain (42%), wrist/hand pain (35%) and shoulder pain (30%). Older age, right-handedness, not currently smoking, emotional exhaustion, belief that musculoskeletal problems are commonly caused by work, and low job security were the statistically significant risk factors for MSP in different anatomical sites.

**Conclusions:**

A high prevalence of MSP in the neck, low back, wrist/arm and shoulder was observed among Estonian computer users. Psychosocial risk factors were broadly consistent with those reported from elsewhere. While computer users should be aware of ergonomic techniques that can make their work easier and more comfortable, presenting computer use as a serious health hazard may modify health beliefs in a way that is unhelpful.

## Background

In recent decades, occupational use of information technology has increased dramatically. In many countries, computer work is widely perceived as a new risk factor for musculoskeletal disorders (MSDs), which collectively have become the most frequently diagnosed occupational diseases in Estonia [1] and other European countries [2].

Associations between computer work and MSDs have been demonstrated in several studies, with reported 12-month prevalence rates of musculoskeletal pain (MSP) in the neck, back and upper extremities of 55-69%; 31-54%; and 15-52% [3-6].

Risk factors for MSP include demographic (gender, age) and other personal characteristics (height, smoking, tendency to somatise), and also psychosocial, organizational and physical aspects of work (duration of computer work, computing skills, awkward postures, repetitive movements, scope for regular breaks and performance of exercises) [7-11].

Until this study, the prevalence and determinants of MSP among computer users had not been studied in Estonia. The study contributes this data to the literature.

The aim of this study was to assess the prevalence of MSP by anatomical region and to investigate the association of MSP with work-related risk factors and personal characteristics among the computer users at two major universities in Estonia.

## Methods

### Study design and subjects

The research formed part of a larger investigation, the Cultural and Psychosocial Influences on Disability (CUPID) study, coordinated by the University of Southampton (UK). The CUPID study involves 18 countries, and explores the contribution of cultural and socioeconomic factors to the occurrence of MSDs and associated disability in different occupational groups, including office workers using computers. Details of data collection and various characteristics of the full CUPID study sample have been published previously [12].

As part of the CUPID study, a cross-sectional survey was carried out during October to November 2008 among office workers at the University of Tartu (n = 315) and the Estonian University of Life Sciences (n = 100). The study sample was assembled from lists provided by The Human Resources Departments. To be included, subjects had to meet the following criteria: use of computer keyboard for at least 4 hours per day; age 20-59 years; and length of employment in the current job at least 12 months. The questionnaires were sent to the computer users via inner post. Filled questionnaires were sent back also via inner post in sealed envelopes.

The study was approved by the Ethics Committee on Human Research University of Tartu (Prot. No. 173/T-14 18.08.2008). Participation in the survey was voluntary and all the participants provided written informed consent.

### Variables

The self-administered questionnaire was used to ascertain the occurrence of MSP lasting for longer than one day in the past 12 months at different anatomical sites, its impact (e.g. medical consultation, sickness absence), and various possible risk factors. Many of the variables have been described in detail previously [12]. In brief there were questions about demographic characteristics; education; height; smoking habits; current occupation; pain in different anatomical regions and associated disability for tasks of daily living; awareness of others with musculoskeletal pain; fear-avoidance beliefs concerning upper limb and low back pain; awareness of repetitive strain injury or similar terms; distress from common somatic symptoms; mental health; and sickness absence in the past 12 months because of musculoskeletal problems and other types of illness. Participants were asked whether during the past 12 months they had experienced pain in each of six anatomical regions - low back, neck, shoulder, elbow, wrist/hand and knee, illustrated in diagrams. Pain at an anatomical site was considered frequent if during the past 12 months it had been present for longer than 30 days in total. The questions about pain had been used successfully in earlier studies [13-16]. The risk factors for MSP that were examined divided into two groups - individual characteristics and aspects of work. The first group included gender, age, handedness, smoking habits*,* somatizing tendency, mental health and health beliefs about musculoskeletal pain. Somatizing tendency was classified according to the number of physical complaints from a total of five (faintness or dizziness; pains in the heart or chest; nausea or upset stomach; trouble getting breath; and hot or cold spells) that had been distressing in the past week, and was classified to three levels: no complaints; one complaint; and two or more complaints. Questions were taken from the Brief Symptom Inventory [17].

Two aspects of mental health were assessed – emotional status (mood) and burnout. Mood was scored using questions from the relevant section of the SF-36 questionnaire [18], and classified to three levels with cut-points at one standard deviation below and above the mean for the study sample. An Estonian translation of the Maslach Burnout Inventory (MBI) [19] was used to measure burnout. This comprises 22 items in three subscales (emotional exhaustion (9 items), depersonalization (5 items) and personal accomplishment (8 items)), each of which is graded using a 7-point Likert-type scale (range 0-6). Summed scores were obtained for each subscale, and were classified to three levels, again with cut-points at one standard deviation below and above the mean for the study sample. The MBI was used only in the Estonian arm of the CUPID study.

Health beliefs were assessed in three domains: fear avoidance, prognosis and work attribution. Fear avoidance was classed as present if the respondent completely agreed both for someone with back pain and for someone with arm pain, that physical activity should be avoided as it might cause harm and that rest is needed to get better. The questions on fear-avoidance beliefs were adapted from the Fear Avoidance Beliefs Questionnaire [20]. Beliefs about prognosis were classed as pessimistic if the respondent completely disagreed that these musculoskeletal problems usually get better within three months and completely agreed that neglecting problems of this kind can cause permanent health problems. Work attribution was deemed to occur if the respondent completely agreed that both back and arm pain are commonly caused by work.

The work-related risk factors that were assessed concerned the organization of work and various psychosocial aspects of employment. The organizational factors were hours of computer keyboard use per day and whether regular breaks were taken. The psychosocial risk factors examined were time pressure, job control, job support, job satisfaction and job security. Time pressure was classed as occurring if the respondent reported piecework or working to complete tasks by a fixed time (deadlines). Job control was considered “high” if the respondent often or sometimes could decide on the sequence of work assignments, as well as how and according to which timetable tasks should be accomplished. Job support was classed as “high” if the respondent could often or sometimes get help and receive support from colleagues or a supervisor/manager. Job satisfaction was “high” if the respondent was satisfied or very satisfied with their job. Job security was classed as “high” if the respondent felt that their employment would be safe or very safe if they suffered a significant illness that had kept them off work for three months.

### Statistical analysis

Data were analysed using Stata version 10.0. Knee and elbow pain were excluded from consideration because knee pain has not been linked with use of computers and the prevalence of elbow pain was relatively low. Descriptive statistics were used to summarize the prevalence of MSP and risk factors. Associations of pain with risk factors were assessed by logistic regression, and summarized by odds ratios (ORs) with 95% confidential intervals (CIs). In the first model MSP were analysed independently, with adjustment only for gender and age. In the second model MSP was mutually adjusted for significant risk factors from the first model (p ≤ 0.05). Only cases with available data on each variable were analysed.

## Results

Questionnaires were sent to the 415 computer users, and 220 of them responded (response rate 53%). However, 18 had to be excluded because they turned out not to meet the eligibility criteria, leaving 202 participants who were suitable for analysis.

Participants were predominantly women (85%) with mean age 40.0 (SD 10.0) years, and most (66%) had been employed in their current job for longer than 5 years. On average, they worked for 40.0 (SD 5.0) hours per week, and used computers in their work for an average of 6.6 (SD 1.5) hours per day. More than half of them (57%) took regular breaks in the course of their work.

The majority of participants (77%) reported MSP affecting at least one of the four anatomical sites (low back, neck, shoulder, wrist/hand) during the past 12 months, including 49 participants (24%) with pain at two sites and 31 participants (15%) with pain at three sites. The low back and neck were the most common sites of pain. Frequent pain, with pain in shoulder and wrist/hand reported less often (Table 1). Within the past 12 months, 81 participants (40%) had consulted a medical practitioner about pain at one or more of the four sites, and 18 (9%) had been absent from work because of such pain.

**Table 1:** Prevalence of musculoskeletal pain among Estonian computer users during the past 12 months by anatomical region

**Anatomical region**	**Pain that lasted longer than a day**		**Frequent pain**		**Pain leading to medical consultation**	
	**N**	**%**	**N**	**%**	**N**	**%**
Low back	84	42	19	9	40	20
Neck	104	51	29	14	50	25
Shoulder	61	30	14	7	22	11
Wrist/hand	70	35	14	7	28	14
Total (N)	319		76		140	

When risk factors for MSP were analysed independently, with adjustment only for gender and age, significantly elevated risks of low back pain were found for somatising tendency, emotional exhaustion, belief that musculoskeletal problems are commonly caused by work, low job support and low job security, while risk was significantly reduced in current smokers (Additional file 1: Table S1). Neck pain was significantly more common in women, at older ages and with somatising tendency and belief that musculoskeletal problems are commonly caused by work. Shoulder pain was significantly associated with emotional exhaustion; and wrist/hand pain with older age, lower odds of left/both-handedness, belief that musculoskeletal problems are currently caused by work and time pressures at work. In mutually adjusted models there were several findings that have 95% confidence intervals with lower limits very close to one, e.g. low job security and low back pain OR 2.29 (0.99, 5.32) and work attribution beliefs and wrist/hand pain OR 2.07 (0.97, 4.45). Despite of associations with most variables were somewhat reduced, but female sex remained significantly associated with neck pain, older age with wrist/hand pain, left/both-handedness with lower odds of wrist/hand pain, emotional exhaustion with low back and neck pain, belief that musculoskeletal problems are commonly caused by work with low back and neck pain, and time pressure with wrist/hand pain (Additional file 1: Table S1). In addition, low job support carried a low risk of neck pain.

When patterns of association were considered across the four anatomical sites, the statistically significant risk factors for MSP were older age (but not for shoulder pain), right-handedness, not currently smoking, emotional exhaustion (but not for wrist/hand pain), belief that musculoskeletal problems are commonly caused by work, and low job security (but not for wrist/hand pain).

## Discussion

This study found a high prevalence of MSP, especially in the neck and low back, among Estonian computer users. Various risk factors were identified, and several showed statistically significant associations with pain at all four of the anatomical sites analysed.

The observed 12-month prevalence of neck pain was similar to that reported in New Zealand office workers (51%) [21] and somewhat higher than among a sample of UK office workers (38%) [16]. The prevalence of low back and shoulder pain during the past 12 months was close to that in the other two countries, and the prevalence of wrist/hand pain (35%) was similar to New Zealand (33%) [21], but a little lower than in the UK (44%) [16]. As in several other studies of office workers [3-5], the neck was the most common site of pain.

Not only was the prevalence of reported pain high in our study sample, but as many as 40% of participants had consulted a doctor in the past year because of the problem. This suggests that symptoms were often more than trivial. There could be a range of reasons not to consult with the medical specialist. Estonians are quite closed or introvert by character and usually do not see any reasons to complain about their pain. They will consult a doctor only when the pain becomes more serious or when they are having multiple pain. This is confirmed by the Estonian statistics of occupational diseases. Usually three occupational diseases have been diagnosed on an average patient [22]. In other cases, participants may have felt that medical consultation was unlikely to be useful. Of relevance to this, according to Estonian law, employees do not receive sickness benefit during the first three days of an illness the next five days are paid by employers, and sickness absence beyond that is covered by the Health Insurance Fund [23]. Because of insufficient social security policy in Estonia it is financially adverse to take sick leave. This may influence the participant’s decision to consult a doctor. There are also absence of insurance for occupational accidents and diseases. Furthermore, MSP is not compensated as a work-related disease.

The associations that we found with older age, emotional exhaustion, somatization, belief that musculoskeletal problems are commonly caused by work, and low job security are consistent with results from earlier studies [11,13,24-29].

Contrary to previous reports [24,30], current smokers reported less low back pain than non-smokers. This cannot be explained by smokers taking more frequent breaks from work than non-smokers, since there was no reduction in risk among those who reported regular breaks. It may simply have been a chance occurrence.

Left- or both-handedness was associated with a lower risk of wrist/hand pain. Delisle et al. [31] reported that use of a computer mouse on the left side as compared with the right side of a standard keyboard reduces the extent of motion when moving from the mouse to the keyboard. If such motion causes discomfort, then people who are left-handed may be protected somewhat. However, left- or both-handedness was associated with a lower risk of low back pain, which is difficult to explain biomechanically, and again the observed relationships may have occurred by chance. Also, contrary to previous reports [32], the association with low job support and lower odds of neck pain may have occurred by chance. It may refer to the specific nature of Estonians who are rather modest and introverted. They are trying to deal with their problems on their own rather than asking for help. Bothering others and constant communicating may be more stressful than working by themselves. Participants who do not need so much job support may have less stress and therefore less neck pain.

Our study had various strengths and limitations. It had the advantage of employing validated and widely used questions to ascertain MSP and many of the risk factors. On the other hand, it was limited by its cross-sectional design. The reliability of information regarding the number of days experiencing pain in the past year relies heavily on memory and the other answers may be subjective. The study was also limited the incomplete response from those invited to participate. This raises the possibility of healthy worker selection, response bias and reverse causation. If anything, healthy worker selection would cause the prevalence of MSP to be underestimated. On the other hand, among those invited to participate, workers with pain may have been more inclined to take part, which would have biased prevalence estimates upwards. Furthermore, we cannot exclude the possibility that MSP made workers more likely to report emotional exhaustion and distress from somatic symptoms. We did not address ergonomic risk factors that have been associated with MSP because within our study sample, exposures were rather homogeneous. Nor did we collect information about activities outside work that might have contributed to symptoms.

Despite these limitations, when viewed in the context of other similar investigations [8,9,11,24-28], our study points to risks from psychological factors such as emotional exhaustion, low job security and belief that musculoskeletal problems are commonly caused by work. The participants may come to have these beliefs after they and/or colleagues experienced MSP at work. While computer users should be aware of ergonomic techniques that can make their work easier and more comfortable, presenting computer use as a serious health hazard may modify health beliefs in a way that is unhelpful. A better approach may be to design work so that employees feel valued and supported.

## Conclusion

MSP was prevalent in studied Estonian computer users. Pain was experienced mostly in the neck and low back, and symptoms were associated with specific individual and work-related factors. Further work is needed to confirm our findings in longitudinal studies that are less liable to bias.

## Competing interests

The authors declare that they have no competing interests.

## Authors’ contributions

KO made a substantial contribution to translation of the questionnaire, carried out the data collection and part of the statistical analysis, and drafted the manuscript. EM was the coordinator and principal investigator for CUPID study in Estonia and helped to revise the manuscript. LA contributed to the statistical analysis. MP contributed to the study design and drafting of the manuscript. DC designed the international CUPID study and helped to revise the manuscript. All authors read and approved the final manuscript.

## Pre-publication history

The pre-publication history for this paper can be accessed here:

http://www.biomedcentral.com/1471-2474/15/181/prepub

## Supplementary Material

Additional file 1: Table S1 Associations of musculoskeletal pain in past 12 months with individual characteristics and work-related risk factors. Click here for file
